# An Excellent Clinical Outcome with Stereotactic Radiosurgery in a Geriatric Patient with Multiple and Recurrent Brain Metastases

**DOI:** 10.7759/cureus.1979

**Published:** 2017-12-22

**Authors:** Bicky Thapa, Hamid Borghei-Razavi, Alireza M. Mohammadi, Manmeet Ahluwalia

**Affiliations:** 1 Internal Medicine, Cleveland Clinic, Fairview Hospital; 2 Neurological Institute, Taussig Cancer Center, Cleveland Clinic; 3 Brain Tumor and Neuro-Oncology Center, Cleveland Clinic

**Keywords:** brain metastases, stereotactic radiosurgery, whole brain radiation therapy, geriatric, cognitive, recurrence

## Abstract

The incidence of brain metastases range from 10 to 20% of all adult patients with cancer and lung cancer is associated with one of the highest incidences of brain metastases. In geriatric patients, who already have limited cognitive function, whole brain radiation therapy (WBRT) can be a problem. Stereotactic radiosurgery (SRS) is a one day, outpatient treatment with minimal effect to normal brain and could particularly be useful in elderly patients. We report the case of a geriatric patient with metastatic lung adenocarcinoma who had multiple brain metastases and recurrences, who responded well to the stereotactic radiosurgery (six sessions) with acceptable tumor control.

## Introduction

The brain metastases incidence is actually increasing as there has been an improvement in overall patient survival due to more effective systemic therapy [1-2]. Stereotactic radiosurgery (SRS) is a primary modality of treatment in patient with brain metastases and has demonstrated efficacy in several randomized trials and multicenter studies, whether performed alone or in combination with whole-brain radiation therapy (WBRT) [3-5]. SRS has been shown to be efficacious and safe for a fewer number of brain metastasis. We report an exceptional outcome in a geriatric patient with multiple brain metastases who underwent multiple courses of SRS.

## Case presentation

**Figure 1:**
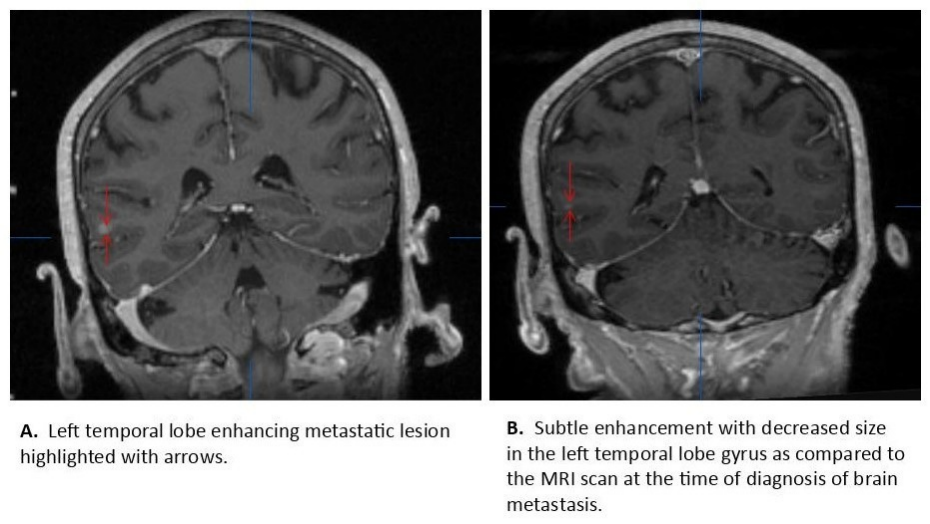
MRI scan of the brain from the brain lab, (A) at the time of the diagnosis or before SRS and (B) after SRS. MRI: Magnetic resonance imaging; SRS: Stereotactic radiosurgery.

An 81-year-old female with medical history significant for Raynaud's syndrome, hyperparathyroidism, and essential hypertension was found to have a subscapular subcutaneous lesion in July 2013, subsequently underwent excision and pathology was reported as a metastatic adenocarcinoma with focal neuroendocrine features in the lesion; molecular marker like epidermal growth factor receptor (EGFR) was negative for mutation and there was no anaplastic lymphoma kinase (ALK) fusion. Computed tomography (CT) chest performed in Aug 2013 revealed a 10 x 18 mm right lower lobe and a separate 10 mm right lower lobe nodule with multiple sub-5 mm nodules throughout both lungs. Positron emission tomography and computed tomography (PET-CT) confirmed fluorodeoxyglucose (FDG) avidity in the right lower lobe as well as three FDG avid pleural lesions and a destructive third lumbar vertebral (L3) lesion. Magnetic resonance imaging (MRI) of the brain (Figure 1) showed a very small lesion in left temporal region for which she underwent SRS.

**Figure 2:**
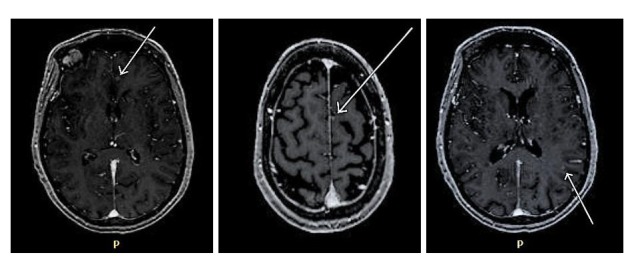
Follow-up MRI of the brain showing new punctate enhancement in the left frontal and parietal region. MRI: Magnetic resonance imaging.

She received carboplatin, pemetrexed, and bevacizumab in October 2013 for systemic disease with excellent initial response. Follow-up MRI of the brain in August 2014 and repeat in October 2014 (Figure 2) demonstrated new multiple brain metastases in left frontal and parietal region for which she underwent SRS in October 2014.

The patient was continued on maintenance pemetrexed until March 2015 and was changed to single-agent paclitaxel for systemic disease. However, paclitaxel failed to control the systemic disease and patient had progression of the systemic disease as well as new multiple brain metastases. In view of the systemic disease progression, the patient was initiated on Nivolumab in June 2015 and had stereotactic radiosurgery in August 2015. However, CT of the chest, abdomen, and pelvis with contrast in March 21, 2016 demonstrated stable disease.

**Figure 3:**
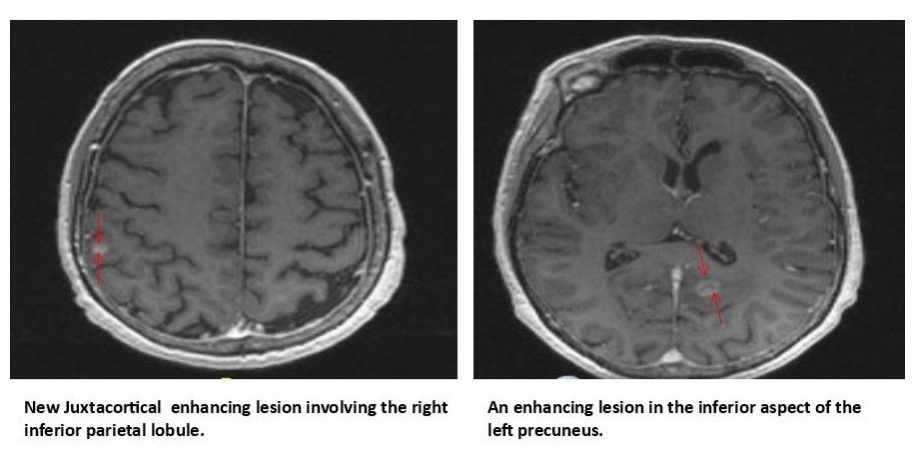
MRI brain with new metastases highlighted by arrows. MRI: Magnetic resonance imaging.

In May 2016 repeat MRI (Figure 3) demonstrated new brain metastases in right parietal region, left precuneus and she again underwent SRS, although CT scans of abdomen and lung on July 2016 again showed stable disease.

**Figure 4:**
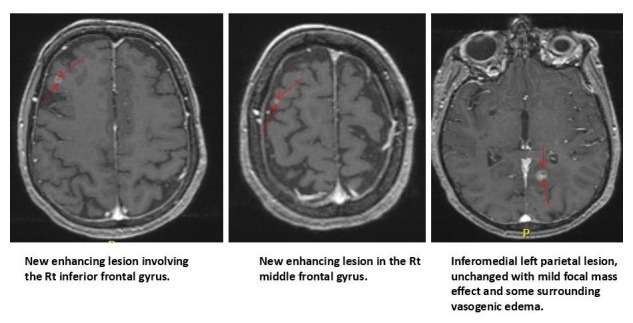
Follow-up MRI brain with new metastases and old lesion. MRI: Magnetic resonance imaging.

MRI brain in July 2016 again revealed new right occipital lesion for which she underwent SRS on July 16. Follow-up MRI brain in September 2016 (Figure 4) revealed two new metastases in right frontal region for which the patient again had SRS.

There was no neurological deficit since the diagnosis of the brain metastases and patient tolerated the SRS treatment fairly well with good quality of the life and with no impairment in neurocognitive function.

The patient has been doing well with stable systemic disease and good performance status with Eastern Cooperative Oncology Group (ECOG) performance score of 0.

There were a total of 12 metastatic lesions identified and treated over three years. They varied in linear size from  3 mm to 12 mm. The characteristics of the brain metastases in the form of target volume, location of the brain metastases and linear size along with radiation doses have been summarized in Table 1.

**Table 1:** Details of brain metastases and radiation dose. SRS: Stereotactic radiosurgery.

Date of SRS	No. of lesions	Location of the lesions	Target volume	Maximum linear size	Dose of radiation
9/26/2013	1	Lt Temporal	0.191 cc	0.8 cm	24 Gy to the 51% local isodose line
10/2/2014	4	Lt Superior frontal	0.015 cc	0.3 cm	24 Gy to the 83% local isodose line
		Lt Parietal	0.071 cc	0.4 cm	24 Gy to the 84% local isodose line
		Lt Anterior frontal	0.047 cc	0.4 cm	24 Gy to the 79% local isodose line
		Lt Mesial frontal	0.071 cc	0.6 cm	24 Gy to the 79% local isodose line
8/10/2015	3	Lt lateral frontal	0.0174 cc	0.3 cm	24 Gy to the 74% local isodose line
		Rt Parietal	0.1989 cc	0.75 cm	24 Gy to the 77% local isodose line
		Lt Occipital	0.642 cc	1.2 cm	24 Gy to the 81% local isodose line
5/27/2016	1	Rt Lateral parietal	0.275 cc	0.9 cm	24 Gy to the 62% local isodose line
7/15/2016	1	Rt Posterior Occipital	0.088 cc	0.6 cm	24 Gy to the 89% local isodose line
9/1/2016	2	Rt Superior frontal	0.05 cc	0.5 cm	24 Gy to the 53% local isodose line
		Rt Lateral frontal	0.111 cc	0.6 cm	24 Gy to the 86% local isodose line

## Discussion

Treatment for brain metastases is challenging and the outcome depends on various factors such as the age of the patient, performance status and the number of metastases, mass affect, tumor location and neurological deficit. Extracranial metastasis and systemic disease control have been a major determinant of the overall survival of the patient with brain metastasis.

Historically, WBRT has been the standard of care for patients with multiple brain metastases but it has also been associated with neuro-cognitive effect [6-7].

SRS has demonstrated efficacy in several randomized trials and multicenter studies, whether performed alone or in combination with WBRT. Recently, SRS has been emerged as the preferred modality of treatment for the patient with multiple brain metastases [8]. SRS has also progressively gained favor because of its minimal impact on the delivery of systemic treatment options and it may also have synergistic effect with novel immunotherapy agents.

Our patient had multiple recurrence of brain metastases since the diagnosis and underwent a total of six courses of SRS within a span of four years. Except for patient’s age (>75 years), she had fairly well controlled systemic disease, good performance status, and no neurological deficit. The patient’s multiple recurrence of the brain metastases has been well controlled with stereotactic radio-surgery with good clinical outcome. In total she received local intracranial treatment with stereotactic radiosurgery for 12 lesions with no neurological deficit. 

The rates of local control have been equivalent between SRS and WBRT but SRS alone has been found to have higher rates of recurrence after treatment as compared to WBRT because SRS only treats the lesion visible on MRI while WBRT also treats microscopic level metastatic disease [9]. Furthermore, overall survival is similar between WBRT and SRS versus SRS alone in randomized studies [9]. Recent studies in patients with brain metastasis have also demonstrated tumor volume as the prognostic factor to predict the clinical outcome [10].

## Conclusions

Stereotactic radiosurgery is a very effective modality of treatment for recurrent and multiple brain metastases depending on the prognostic factors of the patient. Minimal cognitive decline compared with WBRT makes SRS favorable for geriatric patients with limited cognitive capacity. However, more studies are necessary on multiple SRS courses in recurrent brain metastases to determine the neuro-cognitive effect and overall outcome of the patients.
